# Novel strategies in antithrombotic therapy: targeting thrombosis while preserving hemostasis

**DOI:** 10.3389/fcvm.2023.1272971

**Published:** 2023-10-23

**Authors:** Martha M. S. Sim, Semekidus Shiferawe, Jeremy P. Wood

**Affiliations:** ^1^ Department of Molecular and Cellular Biochemistry, University of Kentucky, Lexington, KY, United States; ^2^ Saha Cardiovascular Research Center, University of Kentucky, Lexington, KY, United States; ^3^ Division of Cardiovascular Medicine Gill Heart and Vascular Institute, University of Kentucky, Lexington, KY, United States

**Keywords:** thrombosis, hemostasis, platelet, anticoagulation, thrombin

## Abstract

Antithrombotic therapy is a delicate balance between the benefits of preventing a thrombotic event and the risks of inducing a major bleed. Traditional approaches have included antiplatelet and anticoagulant medications, require careful dosing and monitoring, and all carry some risk of bleeding. In recent years, several new targets have been identified, both in the platelet and coagulation systems, which may mitigate this bleeding risk. In this review, we briefly describe the current state of antithrombotic therapy, and then present a detailed discussion of the new generation of drugs that are being developed to target more safely existing or newly identified pathways, alongside the strategies to reverse direct oral anticoagulants, showcasing the breadth of approaches. Combined, these exciting advances in antithrombotic therapy bring us closer than we have ever been to the “holy grail” of the field, a treatment that separates the hemostatic and thrombotic systems, preventing clots without any concurrent bleeding risk.

## 1. Introduction

Thrombosis is the pathological formation of a clot within an intact vessel, which blocks blood flow, resulting in an ischemic injury. Depending on their location, thrombi can be the direct cause of life-threatening events, such as myocardial infarctions, pulmonary emboli, and strokes (1). Thus, safe and effective antithrombotic therapies are a critical component of our medical system. However, all existing antithrombotics carry a risk of bleeding, which can also be life-threatening. This is because the same components responsible for thrombus formation (blood platelets and coagulation) are also necessary for hemostasis, the healthy formation of a blood clot in response to vessel injury (2). Therefore, research is ongoing to identify new targets in this system, which may provide safer treatment. Here, we summarize the current therapeutic strategies, targeting platelets and coagulation factors, and discuss the new approaches that are in development, emphasizing the diversity of strategies and targets being evaluated.

## 2. The present and future of antiplatelet therapeutics

Antiplatelet therapy has become a fundamental component in the treatment of cardiovascular disease. At the site of vascular injury or in response to vascular pathology, such as rupture of an atherosclerotic plaque, platelets can activate and aggregate intravascularly, typically as arterial thrombi (1). Similarly, platelet activation and aggregation are associated with inflammatory conditions, and platelet depletion with the development of disseminated intravascular coagulopathy, in conditions such as sepsis (3). Antiplatelet drugs are intended to prevent or limit platelet activation and aggregation and are generally used in acute coronary syndrome (ACS) and ischemic stroke patients for long-term control or secondary prevention (4–7). As the same mechanistic processes are responsible for both physiological and pathological platelet aggregation, striking a balance between the beneficial and harmful effects of antiplatelet therapy continues to be a challenge. In practice, this is clinically managed by careful consideration of optimal therapeutic regimens and duration of therapy, while prioritizing treatment for patients whose thrombotic risk clearly outweighs their risk of bleeding complications. In this section, we discuss the existing and recently developed antiplatelet therapeutics, along with novel strategies that have been proposed based on animal studies.

### 2.1. Current therapeutics

**Figure 1:**
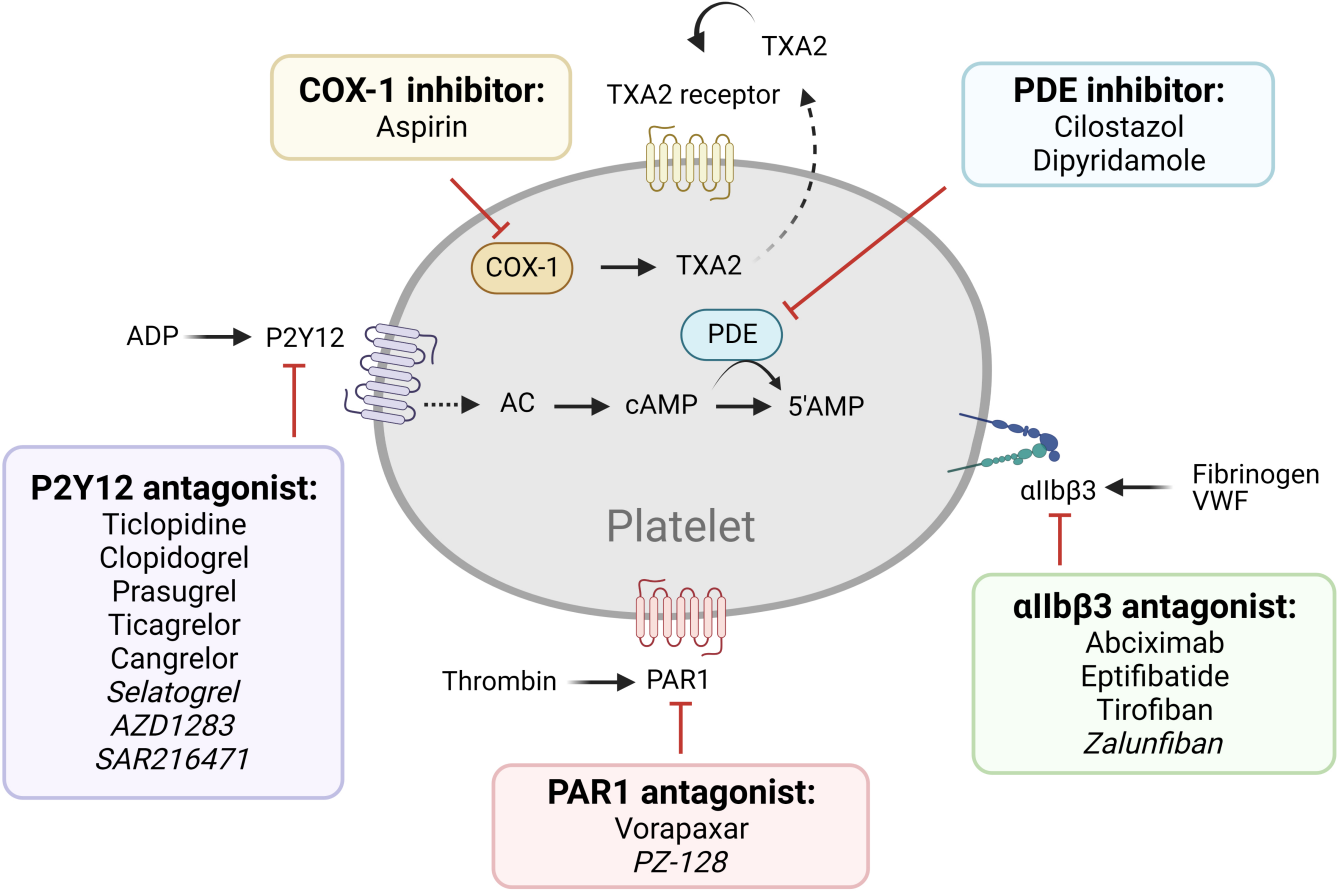
Older and newer generations of antiplatelet therapy targeting established pathways. Commonly used (regular font) and next generation (italicized) antiplatelet therapy of established pathways are listed.

The most commonly used antiplatelet agents target thromboxane A2 (TXA2) and ADP, which are secondary agonists of platelet activation (Figure 1, Table 1). Since the discovery of its antithrombotic effects in 1956, aspirin, used as an anti-inflammatory agent, has been one of the most important antiplatelet agents (8). Aspirin acts by irreversibly inhibiting cyclooxygenase-1 (COX-1), limiting the access of arachidonic acid to its active site and preventing prostaglandin G2 and H2 synthesis, and subsequent TXA2 production (9). Aspirin also inhibits COX-2, which is expressed by ∼10% of circulating platelets, though at ∼170-fold lesser potency compared to COX-1 (10). Its full antiplatelet effect is reached at a low dose of 75–100 mg/day, whereas COX-2 inhibitory effects are mainly observed with higher doses of >500 mg/day, which may result in side effects such as gastrointestinal bleeding, without additional benefit to the antiplatelet effect (11). Apart from its effects on platelet activation, aspirin also acetylates lysine residues on fibrinogen, enhancing fibrin clot permeability and lysis, and therefore, results in less stable clots (12).

**Table 1:** Current antithrombotic therapy.

Target	Name	Type	Administration	Status (indications or developmental stage)
Current and *newer generation* antiplatelet therapy				
COX-1	Aspirin	Small molecule	Oral	ACS, CAD, PAD, CVD, Stroke, TIA
P2Y12	Clopidogrel Prasugrel Ticagrelor Cangrelor *Selatogrel* *AZD1283* *SAR216471*	Small molecule Small molecule Small molecule Small molecule Small molecule Small molecule Small molecule	Oral Oral Oral IV SC Oral Oral	ACS, CAD, CVD ACS with PCI ACS ACS with PCI Phase III Preclinical Phase II
PDE	Cilostazol Dipyridamole	Small molecule Small molecule	Oral Oral	PAD Stroke, TIA
PAR1	Vorapaxar *PZ-128*	Small molecule Pepducin	Oral IV	PAD Phase I
αIIbβ3	Abciximab Eptifibatide Tirofiban *Zalunfiban*	Chimeric h-mFab Cyclic peptide Small molecule	IV IV IV SC	ACS with PCI ACS with PCI ACS with PCI Phase III
Current anticoagulation therapy				
Vitamin K Cycle	Warfarin and others	Small molecule	Oral	Prevention of thrombotic events in high-risk patients
Factor Xa, Thrombin (through binding antithrombin)	Heparins Danaparoid	Polysaccharide Heparinoid	IV, SC IV, SC	Treatment and prevention of VTE, thrombus prevention in AF, treatment of DIC
Factor Xa	Fondaparinux (through binding antithrombin) Rivaroxaban Apixaban Edoxaban Betrixaban	Pentasaccharide Small molecule Small molecule Small molecule Small molecule	SC Oral Oral Oral Oral	Treatment and prevention of VTE, thrombus prevention in AF, alternative treatment of HIT
Thrombin	Hirudins Argatroban Dabigatran	Peptide Small molecule Small molecule	IV IV Oral	Treatment and prevention of VTE and ACS, thrombus prevention in AF, HIT

COX-1, cyclooxygenase-1; ACS, acute coronary syndrome; CAD, coronary artery disease; PAD, peripheral artery disease; CVD, cardiovascular disease; TIA, transient ischemic attack; PCI, percutaneous coronary intervention; PDE, phosphodiesterase; PAR, protease-activated receptor; h-mFab, chimeric human-murine antibody fragment of IgG; IV, intravenous; SC, subcutaneous; VTE, venous thromboembolism; AF, atrial fibrillation; DIC, disseminated intravascular coagulation; HIT, heparin-induced thrombocytopenia.

P2Y12 antagonists are another category of main-line antiplatelet therapeutics. The thienopyridines class consists of ticlopidine, clopidogrel, and prasugrel; and the nucleoside-nucleotide derivatives include ticagrelor and cangrelor. Thienopyridines are prodrugs which require hepatic cytochrome P-450 (CYP450)-dependent metabolism (13). Clopidogrel blocks the P2Y12 receptor irreversibly by modifying a cysteine residue (14, 15). On the other hand, ticagrelor and cangrelor do not require liver-dependent metabolism and are reversible, competitive P2Y12 inhibitors (16). P2Y12 inhibitors are often used for patients with CAD after percutaneous coronary intervention (PCI) in combination with aspirin (dual antiplatelet therapy/ DAPT) and for patients with ACS, with or without PCI, with guidelines recommending varying durations for secondary prevention (17). Due to genetic factors involved in CYP450 metabolic pathways, clopidogrel shows widely variable inhibition of platelet activation, with ∼30% of treated individuals categorized as poor to intermediate responders to the drug (18). Genotype-guided strategies in clopidogrel therapy have been successful (19), suggesting value in individualized pharmacogenetics as a treatment strategy in clinical practice (18). Recently, the more potent and predictable ticagrelor and cangrelor have seen increasing use (20).

Other current antiplatelet therapy (Figure 1) includes phosphodiesterase (PDE) inhibitors (cilostazol, dipyridamole), αIIbβ3 antagonists (abciximab, eptifibatide, and tirofiban), and protease-activated receptor-1 (PAR1) antagonists (vorapaxar) (4–7). PDE inhibitors reduce platelet reactivity by increasing the cyclic nucleotides cAMP and/or cGMP, thereby dampening cytoskeletal rearrangement, integrin αIIbβ3 activation, and platelet secretion by interfering with activation signaling pathways (21). The combination of aspirin-dipyridamole is used for secondary prevention of cerebrovascular atherothrombotic events (22, 23). The αIIbβ3 antagonists prevent platelet aggregation by selectively blocking the fibrinogen receptor (24). Integrin αIIbβ3 can be targeted by a chimeric human-murine monoclonal antibody (abciximab), a synthetic cyclic heptapeptide based upon a sequence found in the snake venom platelet inhibitor disintegrin (eptifibatide), or an RGD-based peptidomimetic analog that specifically binds to αIIbβ3 on resting platelets (tirofiban) (24). The PAR1 antagonists block platelet activation by thrombin, the most potent agonist generated at vascular injury or plaque rupture sites via coagulation activation (25). Despite their great promise, however, several clinical trials have indicated safety concerns including elevated risk of major bleeding (26, 27).

Overall, antiplatelet therapy is still associated with non-negligible to high bleeding risk and thrombocytopenia, a meaningful variability in individual response due to genetic factors, and generally poor biological response in patients with comorbidities, such as diabetes and obesity (4–7, 18). In the next section, pharmacological approaches to platelet inhibition currently being considered, under development, or undergoing clinical testing, will be reviewed.

### 2.2. New generation of drugs targeting established pathways

Several new antiplatelet agents, which target the pathways described above, are currently in clinical trials (Figure 1). These include:

(1) New P2Y12 antagonists: Selatogrel is a selective, potent, and reversible platelet P2Y12 antagonist, with the advantage of subcutaneous administration, allowing self-dosing and usage in the emergency setting of ACS or unconscious patients (28, 29). A Phase III clinical trial is ongoing. Other highly potent P2Y12 inhibitors are under development, including AZD1283 and SAR216471, both of which were associated with higher selectivity, less bleeding, and comparable antithrombotic efficacy compared to ticagrelor in animal models (30, 31). SAR216471 is currently in a Phase II study.
(2) New αIIbβ3 antagonists: Existing receptor antagonists are ligand-mimetics, which may cause a conformation change in αIIbβ3 to a high-affinity state, leading to either paradoxical platelet activation or exposure of ligand-induced binding sites that trigger antibody-mediated platelet clearance and thrombocytopenia in some patients (32, 33). They also are highly potent and associated with a significant increase in bleeding risk, and all require intravenous administration, limiting their utility for long-term therapy. Zalunfiban (RUC-4) is a small molecule inhibitor designed to alleviate this, as it binds to the metal ion-binding site on GPIIIa, maintaining the receptor in the low-affinity state incapable of fibrinogen binding, and therefore, does not induce a conformational change (34, 35). It can also be subcutaneously administered, which would favor its use in urgent settings (34). It showed efficacy in a Phase I study in healthy volunteers and stable CAD patients on aspirin, as it produced high-grade inhibition of ADP-induced platelet aggregation within 15 min of administration with rapid return to normal platelet function within the next 2 h (36). It is currently in a Phase IIb trial. Intracellular inhibitors of αIIbβ3 have also been developed which disrupt integrin activation, preventing the switch to the high-affinity state and subsequent outside-in signaling (37).
(3) New PAR1 antagonists: G protein-coupled receptors (GPCR) are cell surface receptors which upon ligand binding undergo conformational change and activate the associated cytosolic G protein, which further activates an intracellular signaling process. Pepducins are cell-penetrating lapidated peptides that are designed to selectively target the intracellular receptor-effector interface of a GPCR, by conjugating the intracellular loop portion of the receptor, including PAR1 (38). Existing PAR1 inhibitors interfere with both prothrombotic and cytoprotective downstream pathways, while the pepducin technology allows for selective control of downstream signaling pathways (39). PZ-128 is a pepducin inhibitor of PAR1 proposed for CAD treatment that has completed a Phase II clinical trial, showing that it was well tolerated in ACS patients undergoing PCI and did not cause bleeding even when administered on top of DAPT and heparin (40).

### 2.3. New antiplatelet therapy directed against proposed target pathways

**Figure 2:**
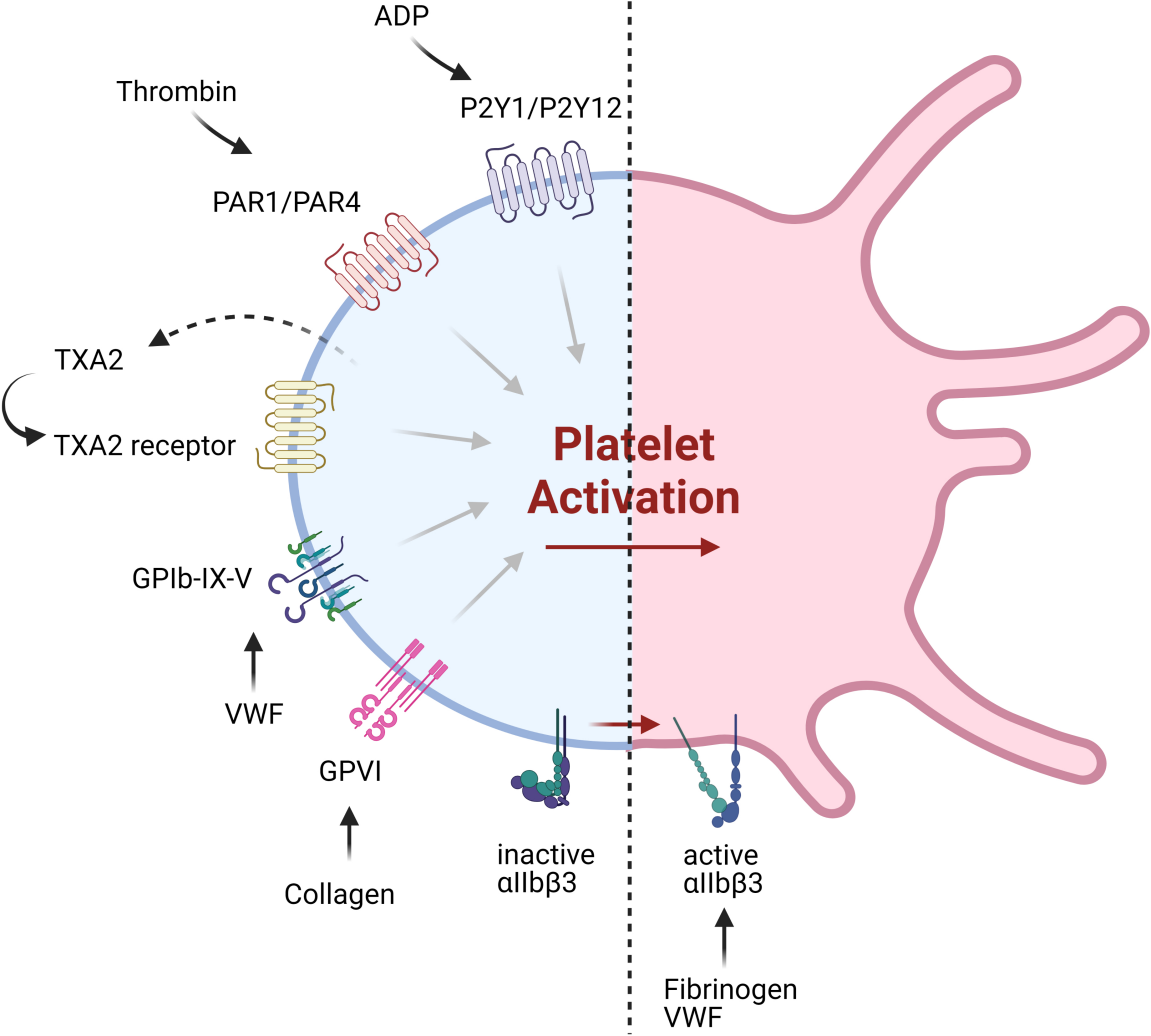
Current and emerging targets of antiplatelet therapeutics. Shown are various platelet receptors implicated in major pathways of platelet activation, which are being targeted by currently available antiplatelet therapy or those under development.

In addition to the development of a new generation of drugs targeting recognized pathways, new targets for antiplatelet therapy have also been identified, including platelet receptors and intracellular signaling pathways (Figure 2, Table 2). Many of these are thought to be non-essential for the hemostatic process, and so may provide safer alternatives to the current interventions.

**Figure 3:**
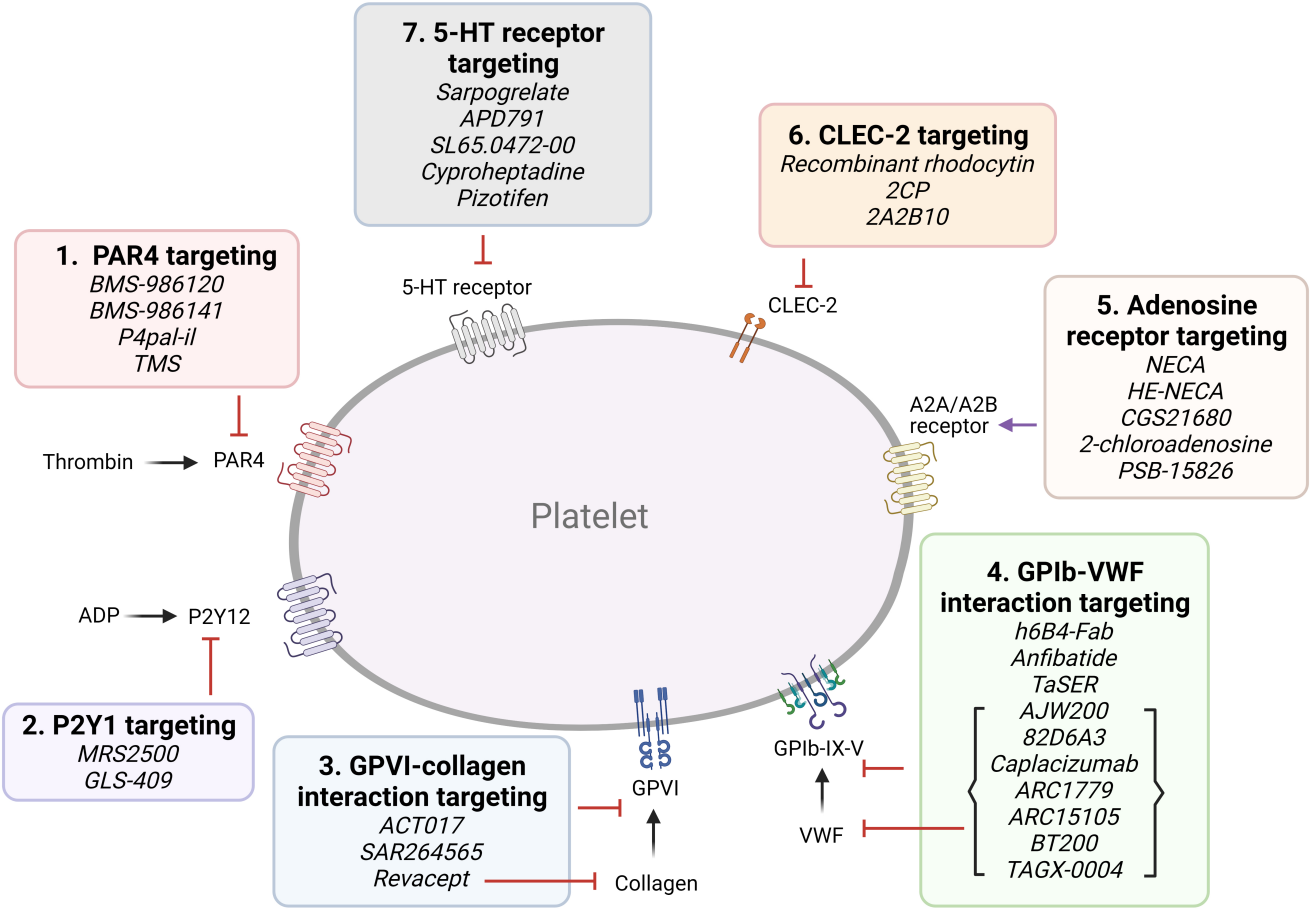
Targeting proposed pathways by modulating platelet receptors. Shown are novel approaches of currently emerging antiplatelet therapy by targeting platelet receptors.

**Figure 4:**
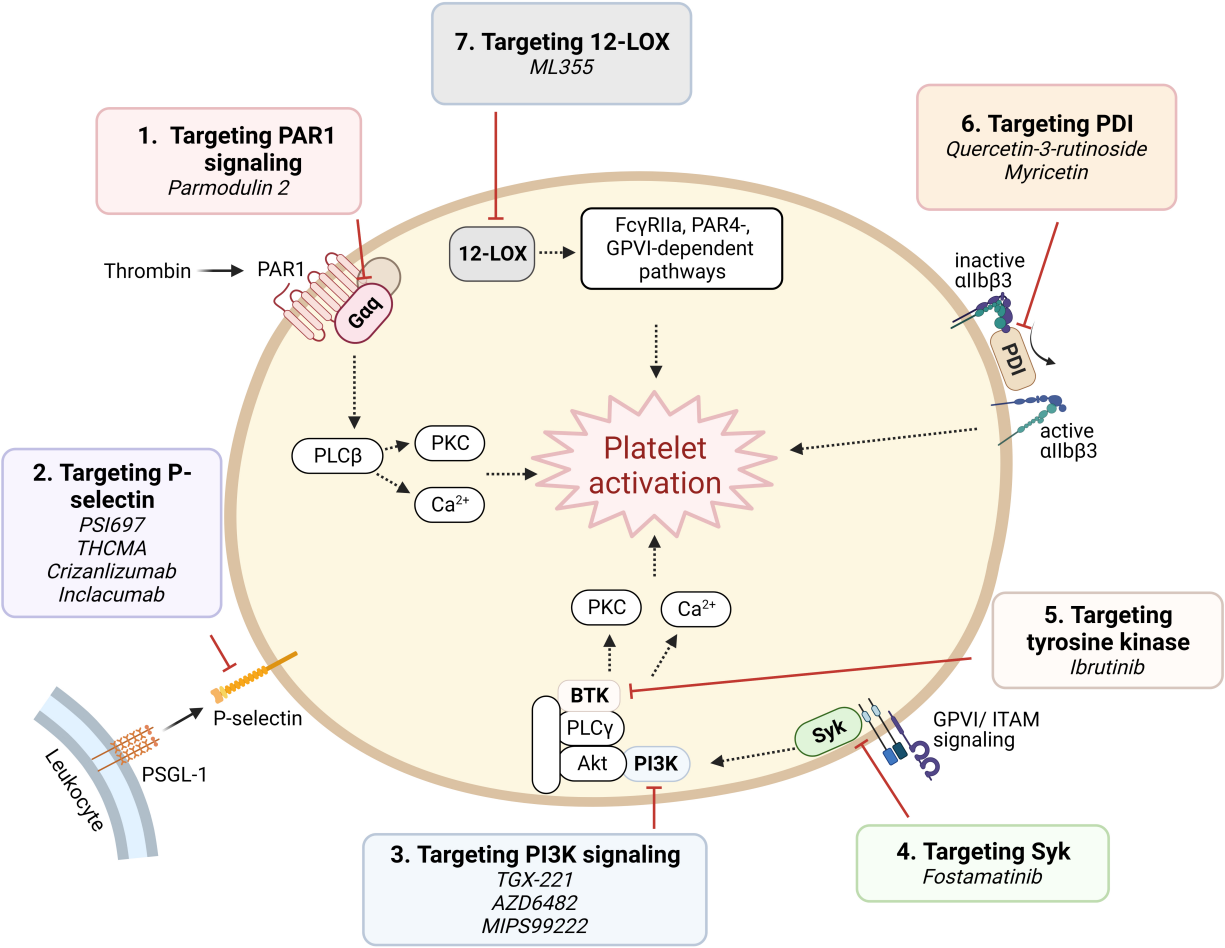
Targeting proposed pathways by modulating signaling component. Shown are novel approaches of currently emerging antiplatelet therapy by targeting platelet signaling component.

A. Targeting platelet surface receptors (Figure 3).
  (1) PAR4 targeting: Both PAR1 and PAR4 are expressed on platelets, and form heterodimers (41). PAR4 activation requires higher thrombin concentrations, and it was proposed to play a more important role in thrombosis than hemostasis (42). BMS-986120 and BMS-986141 are specific, small molecule inhibitors of PAR4, which show antithrombotic efficacy with very low bleeding effect in non-human primates and in healthy individuals, and the latter is showing promise in an ongoing clinical trial (42, 43). P4pal-i1 is a PAR4 pepducin inhibitor currently being investigated for antithrombotic properties, and has been shown to significantly decrease arterial occlusion in guinea pigs (44). And lastly, 3,5,2′,4′-tetramethoxystilbene (TMS) is a fully methylated analog of resveratrol, a phenol found in red wine. TMS binds PAR4 and has been shown to reduce thrombus formation *in vitro* (45).
  (2) P2Y1 targeting: Platelets express P2Y1 and P2Y12 ADP-receptors (15, 46). MRS2500, an adenosine analog developed as a specific P2Y1 antagonist, has been shown to provide strong protection against systemic thromboembolism upon intravenous injection of collagen and adrenalin in mice, while only moderately prolonging bleeding time (47), and exhibited antithrombotic effects in cynomolgus monkeys (48). GLS-409, an analog of the naturally occurring compound adenosine tetraphosphate, inhibits ADP-induced platelet aggregation, and significantly inhibits thrombosis in animal models, with minimal increase in bleeding time (49). To date, there is no clinical trial assessing the P2Y1 antagonists.
  (3) Glycoprotein VI (GPVI) targeting: GPVI is the major collagen receptor on platelets (50). Glenzocimab (ACT017) is a humanized monoclonal fragment antigen-binding (Fab) domain against platelet GPVI, which has been shown to inhibit aggregation and procoagulant activity on collagen-stimulated platelets, as well as adhesion and thrombus formation on collagen surfaces *in vitro* (51, 52). A Phase II/III trial is currently being conducted to evaluate its efficacy and safety in acute ischemic stroke and a Phase IIb in treating myocardial infarction is planned for the near future. SAR264565 is another humanized Fab directed against GPVI, which potently inhibits collagen-induced platelet aggregation (53). Revacept, on the other hand, is a soluble dimeric GPVI-Fc fusion protein containing the fragment crystallizable (Fc) portion of human IgG1, and was developed to specifically bind fibrillar collagen (54). Revacept binds collagen, preventing interaction with circulating platelets and von Willebrand factor (VWF), and resulting in antithrombotic effects. As it does not directly bind platelets, it does not interfere with platelet activity or cause thrombocytopenia (54, 55). It has recently completed a Phase II clinical trial (56, 57). Recently, new selective GPVI antagonists have been identified from large chemical database screening as reported by Olğaç et al. (58), which showed promising antithrombotic properties *in vitro*. Finally, nanobodies raised against the extracellular domain of GPVI have emerged as potential therapeutics (59).
  (4) GPIb-VWF interaction targeting: GPIb*α* is a central component of the GPIb-IX-V complex expressed on platelets, which binds to VWF (60). VWF binding to collagen and its unfolding under higher shear rates exposes binding sites to platelet GPIb and mediates platelet adhesion and thrombus formation (61). Several GPIb-blocking antibodies have been developed and tested for their antiplatelet effects, such as the monoclonal antibody h6B4-Fab, which inhibits ristocetin-induced platelet aggregation in non-human primates, with only a mild prolongation of skin bleeding time (62, 63). Anfibatide is a C-type lectin (CLEC) purified from snake venom, which inhibits the binding of VWF and *α*-thrombin to GPIb*α* (64). It showed strong inhibition of murine and human platelet thrombus formation at low and high shears in *ex vivo* experiments (65), with a Phase I study in healthy volunteers showing good inhibitory effects without significant prolongation in bleeding time. Serine protease inhibitor (SERPIN)-based fusion proteins have also been proposed as antiplatelet therapy (66). A fusion protein called TaSER (targeted SERPIN), consisting of a variable heavy domain of heavy chain (VHH) with function-blocking activity against GPIb*α* and a thrombin-specific SERPIN, has been shown to block VWF binding and limit thrombus formation (67). Various monoclonal antibodies directed against VWF are also being pursued as antiplatelet therapy. AJW200 is a humanized IgG4 monoclonal antibody against the A1 domain of VWF, whereas 82D6A3 is directed against the A3 domain (68, 69). They both show promise in animal models and the former has completed a Phase I study, showing therapeutic promise without prolonging the skin bleeding time. ALX-0081 is a first-in-class, bivalent humanized nanobody directed against the A1 domain of VWF (70). The bivalency allows for high-affinity interaction with VWF-A1, leading to potent inhibition. Preclinical studies in cynomolgus monkeys showed potency in inhibiting ristocetin-induced platelet aggregation with 1.6- and 6-fold less prolongation of bleeding time compared to clopidogrel and abciximab, respectively (70), while studies in a guinea pigs thrombotic stroke model demonstrated reduction in brain infarct size (71). Caplacizumab, derived from ALX-0081, was approved in Europe in 2018 and in the USA in 2019 for use in acquired thrombotic thrombocytopenia purpura (TTP) (72). However, after an inconclusive Phase II trial, drug development for atherothrombotic indications was discontinued (73). Finally, nucleic acid aptamer technology has also been pursued. Aptamers are single-strand DNA or RNA molecules that form 3D structures which specifically bind to their target protein. They are potentially superior to antibodies due to their manufacturing cost, specificity, small size, lack of immunogenicity, and ease of reversal. ARC1779 is a nuclease-resistant aptamer designed to bind to VWF-A1, inhibiting VWF-dependent platelet aggregation (74). ARC15105 is a second generation VWF-A1 aptamer with improved potency and pharmacokinetics (75). BT200, derived from ARC15105, has been successfully tested in healthy volunteers and in a Phase IIa trial (76, 77). More recently, a novel DNA aptamer TAGX-0004, which targets VWF-A1 with very high affinity and specificity was generated (78). TAGX-0004 contains a unique mini-hairpin DNA structure which confers further resistance to nuclease degradation thus extending its half-life *in vivo*. It showed superior thrombus inhibition to ARC1779 and comparable to caplacizumab (78).
  (5) Adenosine A2A and A2B receptor targeting: Adenosine is a purine metabolite in plasma resulting from ecto-50-nucleotidase activity (7). It has a very short (∼1 s) half-life due to enzymatic conversion and has been used widely as an antiarrhythmic agent (7). Platelets express adenosine GPCR receptors, and their activation leads to inhibition of platelet activation and aggregation by increasing the cellular cAMP level (79). Several adenosine receptor agonists are being evaluated as antiplatelet agents. Previously evaluated existing adenosine receptor agonists include 5′-N-ethylcarboxamidoadenosine (NECA), HE-NECA, CGS 21680, 2-chloroadenosine, and PSB-15826, which were shown to possess antiplatelet effects *in vitro*, and antithrombotic effects *in vivo* (80). However, relatively high doses were required for efficacy, leading to a strong vasodilatory activity and various off target effects.
  (6) C-type lectin-like type II (CLEC-2) targeting: Platelets express CLEC-2, which activates various pathways including platelet activation and thromboinflammation via various endogenous ligands such as podoplanin and rhodocytin (81). A recombinant rhodocytin, derived from a snake venom protein, was developed and shown to inhibit CLEC-2 in *in vitro* and animal models (82). A small molecule, 2CP, was also reported to act as a chemical inhibitor of CLEC-2 (83). Anti-CLEC-2 mAb 2A2B10 has also been shown to suppress thrombus formation without significant bleeding effects in animal cancer model (84).
  (7) Serotonin/ 5-hydroxytryptamine (5-HT)-receptor interaction targeting: Platelets express 5-HT subtype 2A receptor (85). Serotonin, packaged in dense granules, is a mild platelet activator, and selective serotonin reuptake inhibitors (SSRIs) inhibit 5-HT reuptake by blocking the serotonin transporter (SERT), thereby acting as antiplatelet agents (86). Direct 5-HT2A receptor antagonists are also being investigated for antiplatelet activity. Currently tested SSRIs are MCI-9042 (sarpogrelate) is an SSRI currently being evaluated (87, 88), while several selective small molecule 5-HT2A receptor antagonists are being investigated, including APD791 (temanogrel), SL65.0472-00, and two existing antidepressant cyproheptadine and pizotifen (89–91). Sarpogrelate significantly reduced serotonin and collagen-induced acute pulmonary thromboembolic death in mice (87). In a trial with stroke patients, however, sarpogrelate failed to demonstrate noninferiority to aspirin, although it was associated with a reduced rate of bleeding complications (88).
B. Targeting platelet signaling components (Figure 4).
  (1) Targeting signaling downstream of PAR1: Parmodulins are novel small molecule allosteric inhibitors of PAR1 which act on cytosolic Gαq subunit signaling downstream of the receptor, inhibiting integrin activation and platelet aggregation. Parmodulin 2, the most selective compound developed, further exhibits anti-inflammatory activity through PAR1 signaling inhibition in endothelial cells, in mouse models (92, 93). No parmodulin clinical trials have started yet.
  (2) P-selectin targeting: Platelets store the adhesion molecule P-selectin in *α* granules and express it on the cell surface during activation (94). P-selectin plays a critical role in mediating platelet-leukocyte adhesion, and is therefore implicated in both thrombosis and inflammation (95). Antiplatelet effects of P-selectin inhibition have been recognized by investigations using small molecules or monoclonal antibodies. PSI697, a small molecule P-selectin antagonist, reduced both arterial and venous thrombosis in animals (96), while THCMA, a nanomolecule, was shown to reduce venous thrombosis without inducing bleeding in animal models (97). Crizanlizumab, a humanized IgG2 monoclonal antibody, was approved in 2019 for patients with sickle cell disease with vaso-occlusive symptoms (98). Inclacumab, a monoclonal antibody that reduces myocardial damage in NSTEMI patients treated with PCI without bleeding complications, is currently being investigated in a Phase III trial (99).
  (3) Phosphoinositide 3-kinase *β* (PI3Kβ) targeting: PI3K signaling in platelets plays a major role in the formation of stable shear-dependent αIIbβ3 platelet adhesion (100). Platelets express class I and class II PI3Ks and among these, the class I PI3Kβ plays an important role in platelet activation responses triggered by the P2Y12 activation, GPVI ligation, and by αIIbβ3 outside-in signaling (100). Several PI3Kβ antagonists have been studied and shown to exhibit strong antithrombotic effects without significantly affecting hemostasis (101), including a peptide inhibitor TGX-221, which are consistent with the phenotype of PI3Kβ knockout mice (102). AZD6482, a selective inhibitor of PI3Kβ that blocks its interaction with ATP, is currently in a Phase IIa study (103). MIPS-9922, another selective inhibitor of PI3Kβ, showed promise in mouse studies, while berberine and 2v, its derivative, have been investigated and show antiplatelet aggregation effects (104, 105).
  (4) Spleen tyrosine kinase (Syk)-targeting: Syk is a non-receptor tyrosine kinase important for immunoreceptor tyrosine-based activation motif (ITAM)-dependent platelet activation and in GPVI-induced platelet activation (106). Inhibition of Syk in a mouse model protects against arterial thrombosis without altering bleeding time (106). Fostamatinib is a first-in-class product and the only Syk inhibitor approved by the FDA, and is indicated for treating immune thrombocytopenia (ITP) (107). It provides additional, mild inhibition of platelet aggregation when combined with aspirin and/or ticagrelor (108).
  (5) Tyrosine kinase targeting: Tyrosine kinase inhibitors (TKIs) are widely used as targeted strategies in cancer treatment, with many tyrosine kinases being highly expressed in platelets. Indeed, many TKIs are associated with suppression of platelet activation and mild bleeding. Ibrutinib, a Bruton tyrosine kinase irreversible inhibitor, was shown to reduce platelet adhesion and thrombus formation *in vitro* (109, 110). However, due to the ubiquitous expression of tyrosine kinases throughout the body, off-target effects might be inevitable.
  (6) Protein disulfide isomerase (PDI)-targeting: PDI catalyzes disulfide bond formation and cleavage and acts as chaperone of protein folding (111). PDI is expressed on the surface of resting platelets and secreted upon activation (111). Platelet PDI was shown to regulate thrombus growth without affecting platelet adhesion or fibrin generation in an arterial injury mouse model. Anti-PDI antibodies and bacitracin, a nonselective PDI inhibitor, reduced thrombus formation and fibrin generation (112). As PDI is highly ubiquitous in tissue distribution, and furthermore, available PDI inhibitors are non-selective and often cytotoxic, off target effects remain a challenge in drug development. Recently, however, isoquercetin and myricetin, members of the flavonoid family, have gained interest and are being investigated as antiplatelet agents, with promising results in a Phase II clinical trial for the former (113, 114).
  (7) 12-Lipoxygenase (12-LOX) targeting: 12-LOX is an oxygenase predominantly expressed in platelets, participates in arachidonic acid metabolism, producing downstream eicosanoids which are involved in platelet activation and proinflammatory response. In platelets, 12-LOX plays a role in αIIbβ3 activation and FcγRIIa-, PAR4-, and GPVI-mediated platelet activation (115, 116). ML355 is a potent and selective 12-LOX inhibitor, which has been investigated as an antiplatelet and shown promising results. ML355 inhibited platelet adhesion and thrombus formation under flow *in vitro*, and similarly reduced thrombus formation in mice without significantly affecting normal hemostasis function (117).

**Table 2:** Novel antithrombotic therapy.

Target	Name	Type	Administration	Status (developmental stage)
Emerging antiplatelet therapy: targeting platelet surface receptors				
PAR4	BMS-986120 BMS-986141 P4pal-i1 TMS	Small molecule Small molecule Pepducin Small molecule	Oral Oral IV IV	Phase I Phase II Preclinical Preclinical
P2Y1	MRS2500 GLS-409	Small molecule Small molecule	IV IV	Preclinical Preclinical
GPVI-collagen	ACT017 SAR264565 Revacept	hMoAb hFab fusion protein	IV IV IV	Phase II/III Preclinical Phase II
GPIb-VWF	h6B4-Fab Anfibatide TaSER AJW200 82D6A3 Caplacizumab ARC1779 ARC15105 BT200 TAGX-0004	hMoAb Small molecule Small molecule hMoAb MoAb h-nanobody DNA aptamer DNA aptamer DNA aptamer DNA aptamer	IV IV IV IV IV IV IV SC SC N/A	Preclinical Phase II Preclinical Phase I Preclinical Phase II Phase I Preclinical Phase II Preclinical
Adenosine receptor	NECA HE-NECA CGS21680 2-chloroadenosine PSB-15826	Small molecule Small molecule Small molecule Small molecule Small molecule	N/A IV N/A N/A N/A	Preclinical Preclinical Preclinical Preclinical Preclinical
CLEC-2	rRhodocytin 2CP 2A2B10	Small molecule Small molecule MoAb	N/A N/A N/A	Preclinical Preclinical Preclinical
5-HT receptor	Sarpogrelate APD791 SL65.0472-00 Cyproheptadine Pizotifen	Small molecule Small molecule Small molecule Small molecule Small molecule	Oral IV Oral Oral Oral	Preclinical Phase II Preclinical Preclinical Preclinical
Emerging antiplatelet therapy: targeting platelet signaling components				
PAR1 signaling	Parmodulin 2	Parmodulin	IV	Preclinical
P-selectin signaling	PSI697 THCMA Crizanlizumab Inclacumab	Small molecule Small molecule hMoAb hMoAb	Oral Oral IV IV	Phase I Preclinical Phase II Phase III
PI3K signaling	TGX-221 AZD6482 MIPS99222	Small molecule Small molecule Small molecule	IV IV IV, oral	Preclinical Phase IIa Phase II/III
Syk	Fostamatinib	Small molecule	Oral	Preclinical
TK	Ibrutinib	Small molecule	Oral	Preclinical
PDI	Isoquercetin Myricetin	Flavonoid Flavonoid	Oral Oral	Phase II/III Preclinical
12-LOX	ML355	Small molecule	Oral	Preclinical
Emerging anticoagulation therapy				
Factor XI	IONIS-FXIRx Abelacimab Osocimab Xisomab Milvexian Asundexian	ASO hMoAb hMoAb hMoAb Small molecule Small molecule	SC IV IV IV, SC Oral Oral	Phase II Phase III Phase II Phase II Phase III Phase III
Factor XII	Garadacimab	hMoAb	SC	Phase III

PAR, protease-activated receptor; TMS, 3,5,2′,4′-tetramethoxystilbene; hMoAb, humanized monoclonal antibody; hFab, humanized antibody fragment of IgG; IV, intravenous; SC, subcutaneous; N/A, not applicable; GPVI, glycoprotein-VI; GPIb, glycoprotein-Ib; VWF, von Willebrand factor; TaSER, targeted serine protease inhibitor; NECA, 5′-N-ethylcarboxamidoadenosine; HE-NECA, 2-hexynyl-NECA; CLEC, C-type lectin; 5-HT, 5-hydroxytryptamine; PI3K, phosphoinositide 3-kinase-β; Syk, spleen tyrosine kinase; TK, tyrosine kinase; PDI, protein disulfide isomerase; LOX, lipoxygenase; ASO, antisense oligonucleotide.

To summarize, several novel approaches to antiplatelet therapy have been eagerly pursued in the last few years, based on our increasing understanding of the mechanisms regulating platelet functions in hemostasis and thrombosis, with many offering promising preclinical results. Although the complete elimination of bleeding risk will probably remain elusive for a while, one or more agents directed against novel targets currently on clinical trial may accomplish this in the not-so-distant future.

## 3. New approaches in anticoagulation

**Figure 5:**
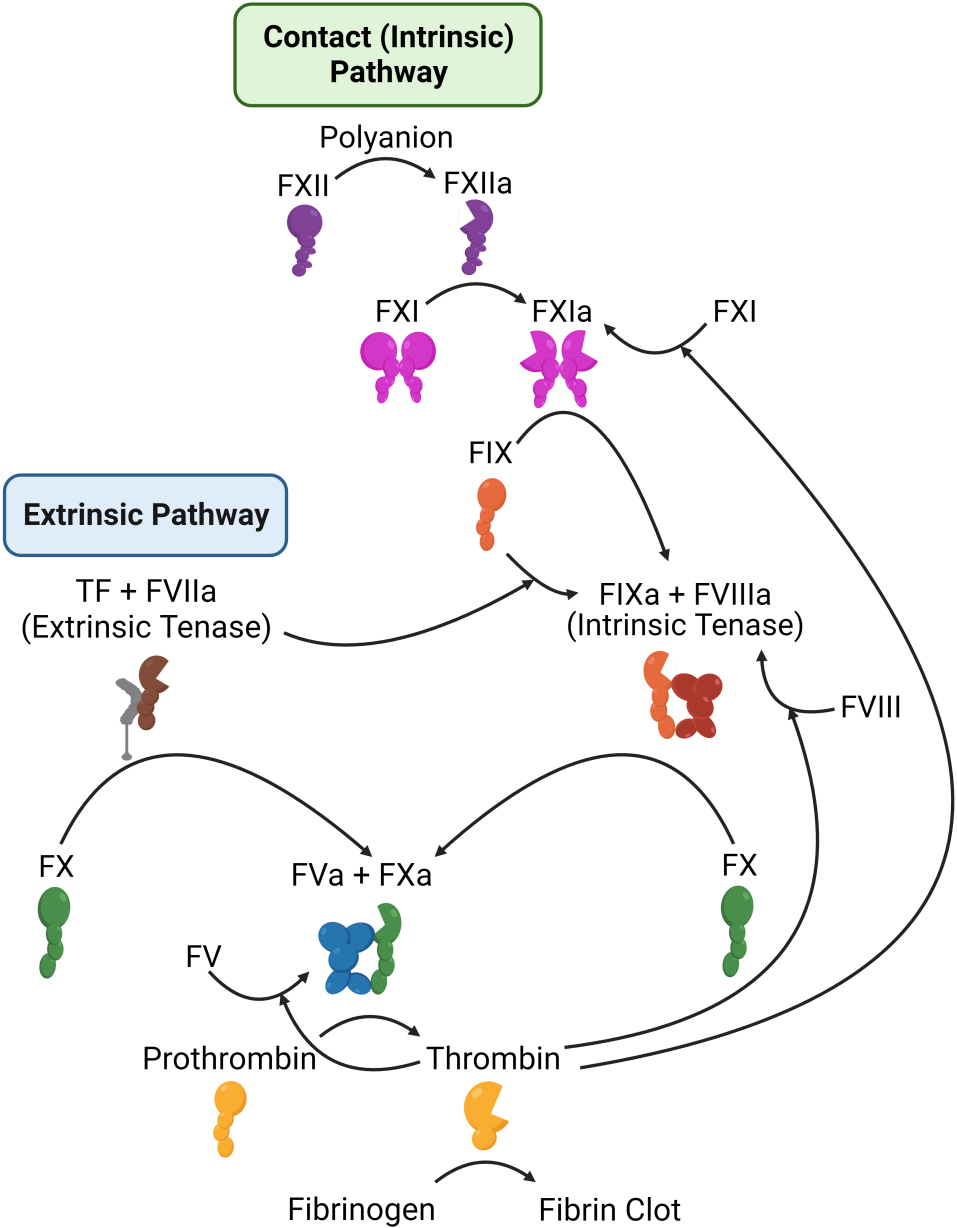
Schematic representation of the coagulation system. Shown are the components of the extrinsic (left) and intrinsic or contact (right) coagulation pathways, along with the mechanisms by which thrombin feeds back to amplify its own activation.

Secondary hemostasis (blood coagulation) (Figure 5) is the process of generating thrombin to convert soluble fibrinogen into insoluble fibrin, in order to stabilize the platelet clot (118). Thrombin is produced through two pathways (termed the extrinsic and intrinsic, or contact, pathways), which culminate in the activation of the serine protease factor Xa (FXa), which then activates thrombin. Classic anticoagulation therapy (Table 1) has focused on inhibition of the final two enzymes in this pathway, factor Xa (FXa) and thrombin, or on a broader inhibition of this pathway. For example, warfarin reduces the availability of Vitamin K, Vitamin K is required to produce γ-carboxylated glutamic acid (Gla) residues, and Gla residues are necessary for the proper folding of multiple coagulation enzymes (119). As a result, factor VIIa (FVIIa), factor IXa (FIXa), FXa, and thrombin are all reduced in warfarin-treated individuals. Heparins have a similarly broad effect, and work by promoting the inhibition of coagulation serine proteases (primarily FXa and thrombin) by plasma antithrombin (120). Due to their potency, warfarin and heparins must be closely monitored. Heparins may be rapidly reversed by protamine, while warfarin is reversed by Vitamin K.

Direct oral anticoagulants (DOACs) have become popular alternatives due to their favorable pharmacological profiles (121). These differ from previous strategies in that they work by specifically inhibiting either FXa (rivaroxaban, apixaban, edoxaban, betrixaban) or thrombin (dabigatran), while leaving other parts of the coagulation system unimpaired.

### 3.1. Reversal of direct FXa and thrombin inhibitors

Though they are safer than warfarin and heparin, direct FXa and thrombin inhibitors do carry risk of bleeding, including potentially fatal bleeds. Therefore, much of the research in this field has shifted towards the development of rapid reversal strategies. One advantage of the broader therapeutics (warfarin and heparins) has been the ease of reversal. Warfarin may be reversed in the short-term by replacement of the missing Vitamin K-dependent proteins, such as through administration of prothrombin complex concentrations or plasma transfusion (122–126) or in the long-term through administration of Vitamin K or by halting the warfarin (125, 127). Negatively-charged heparin is rapidly reversed by positively-charged protamine, though excess protamine itself has anticoagulant and other hazardous effects, so careful dosing is required (128, 129). Other broad anticoagulant reversal agents are also in development. For example, Meijers et al. (130) described OKL-1111, a cyclodextrin-based compound that broadly reverses antithrombotics, including every class of antiplatelet or anticoagulant medication that the authors tested, in a rat tail bleed model. The mechanism of action of OKL-1111 is unknown, though it has direct procoagulant activity in plasma.

One area of recent research has been the creation of rapid reversal agents that are specific for the direct FXa and thrombin inhibitors, which have come in multiple forms:

(1) Monoclonal antibodies: Idarucizumab is a humanized monoclonal antibody Fab fragment targeted against dabigatran, and is able to reverse dabigatran anticoagulant activity within minutes of infusion (131). In addition, idarucizumab does not alter thrombin generation in the absence of dabigatran, suggesting that it does not pose a risk of over-correcting the hemostatic system and promoting thrombosis (132, 133).
(2) FXa mimetics: Andexanet alfa is a recombinant version of FXa in which the membrane-binding Gla domain has been removed and the active site serine residue mutated. Thus, andexanet alfa is catalytically inactive but still able to bind FXa inhibitors, either pharmacologic or endogenous (134). Unlike idarucizumab, andexanet alfa does appear to have some risk of overcorrection, as a recent safety study reported that ∼10% of patients experienced at least one thrombotic event within 30 days of receiving andexanet alfa, unless anticoagulation therapy was restarted (135). This is consistent with *in vitro* work, which indicated that andexanet alfa can increase plasma thrombin generation in the absence of FXa inhibition (136, 137). This effect may be due to the interaction of andexanet alfa with plasma FXa inhibitors, such as antithrombin and tissue factor pathway inhibitor (TFPI), and their neutralization.
  As an alternative approach, Thalji et al. described FXa^I16l^ (138). This single amino acid substitution destabilizes the FXa active site enough that it is resistant to inactivation by plasma inhibitors, such as antithrombin and TFPI, and effectively reverses both rivaroxaban and dabigatran in murine models. FXa^I16l^ has been safe in Phase I and Phase Ib clinical trials (139, 140). Similarly, Verhoef et al. described a FXa homolog found in the venom of *Pseudonaja textilis* (the eastern brown snake), which is resistant to direct FXa inhibitors (141). They utilized this homolog as the basis to design human FXa mutants, with alterations in the substrate binding pocket, which exhibit similar resistance properties. One of these compounds, termed VMX-C001, recently completed a Phase I clinical trial.
(3) Small molecules: Ciraparantag is a small peptide mimetic, which was originally developed to reverse heparins, but found to also reverse DOACs (both thrombin and FXa inhibitors) (142). Ciraparantag functions by directly binding the thrombin and FXa inhibitors and blocking interactions with their respective target proteases. It safely reversed the anticoagulant activities of edoxaban, apixaban, and rivaroxaban in healthy subjects (143, 144).

### 3.2. Contact pathway overview

As summarized above, existing therapies, all of which carry a risk of major bleeding, all target FXa and/or thrombin, either by preventing their production (warfarin) or by blocking their activity. FXa and thrombin lie *at the end* of the coagulation pathway and are both critical for hemostasis. Deficiency in either is very rare and associated with bleeding. Similarly, homozygous deficiency of either is incompatible with life in mice (145–147), and no people have been described with total loss of either protein. To identify coagulation targets that would not adversely impact hemostasis, the focus has shifted instead *to the beginning* of coagulation. Coagulation can be initiated in two ways. First, the extrinsic pathway, consisting of tissue factor and FVIIa, is thought to be the primary initiator of hemostasis. Second, the intrinsic (or contact) pathway is initiated by contact with negatively charged surfaces, leading to factor XII (FXII) activation to FXIIa Meanwhile, high molecular weight kininogen (HK) acts as a bridge, bringing factor XI (FXI) and prekallikrein (PK) close to FXII. Within this cyclic system, FXIIa activates HK-bound PK, leading to the formation of kallikrein, which in turn activates additional FXII. Furthermore, FXIIa also activates FXI in a manner dependent on HK. The subsequent activation of FXIa contributes to the intrinsic coagulation pathway by activating factor IX (FIX), ultimately leading to the generation of thrombin (Figure 5). FXI can be activated independent of the contact system, through a feedback mechanism by thrombin (148, 149), and is thought to be necessary for the amplification of thrombin generation and stabilization of the clot. This may explain why FXII deficiency is not associated with bleeding risk, but FXI deficiency is.

In the laboratory, the contact pathway is activated by exposure of blood/plasma to negatively charged surfaces, such as glass or kaolin, a form of clay. For decades, the physiologic activator of the contact pathway has been unclear. Soil may be a physiologic activator, as the contact pathway may have evolved to recognize and respond to soil that enters the blood through an open wound (150). Many groups, though, have focused on biological polyanions, such as DNA and polyphosphates as potential activators of the coagulation system. DNA is released from activated inflammatory cells in the form of neutrophil extracellular traps (NETs) or the homologous monocyte extracellular traps (METs). NETs and METs are complexes of DNA, histones, and associated proteins, all of which can influence coagulation and platelet function. NETs promote thrombin activation *in vitro*. While there are likely many mechanisms involved, this activity is at least partially dependent on FXII and FXI. In addition, NETs have been shown to bind FXII and promote its activation, and to promote the thrombin-mediated activation of FXI. METs are less studied, compared to NETs, but likely have similar properties.

Polyphosphates are stored within the dense granules of circulating platelets and released upon platelet activation. Similar to NETs, polyphosphates promote FXI activation by thrombin and FXII activation, and promote thrombin generation in a FXII-dependent manner. Also similar to NETs, polyphosphates have contact pathway-independent effects on the coagulation system, such as inhibition of the anticoagulant tissue factor pathway inhibitor and promotion of factor V activation by thrombin. Here, we will discuss strategies that are in development or proposed to target components of the contact pathway, and its activators, as novel anticoagulant agents.

### 3.3. Targeting factors XI and XII

Current evidence favors FXI's role in thrombosis over FXII, with limited support for FXII's involvement in human thrombosis (151). Targeting FXII appears safer as it poses no significant bleeding risk. Solely targeting FXII may not be optimal, however, as thrombin from the extrinsic pathway can activate FXI independent of FXII (148, 149). In contrast, FXI inhibition may cause bleeding, especially in individuals with severe congenital FXI deficiency. FXI inhibition lacks bypass potential but carries off-target risks, like modulating inflammation via inhibiting bradykinin generation, as recently reviewed by Gigante and Ten Cate (152).

**Figure 6:**
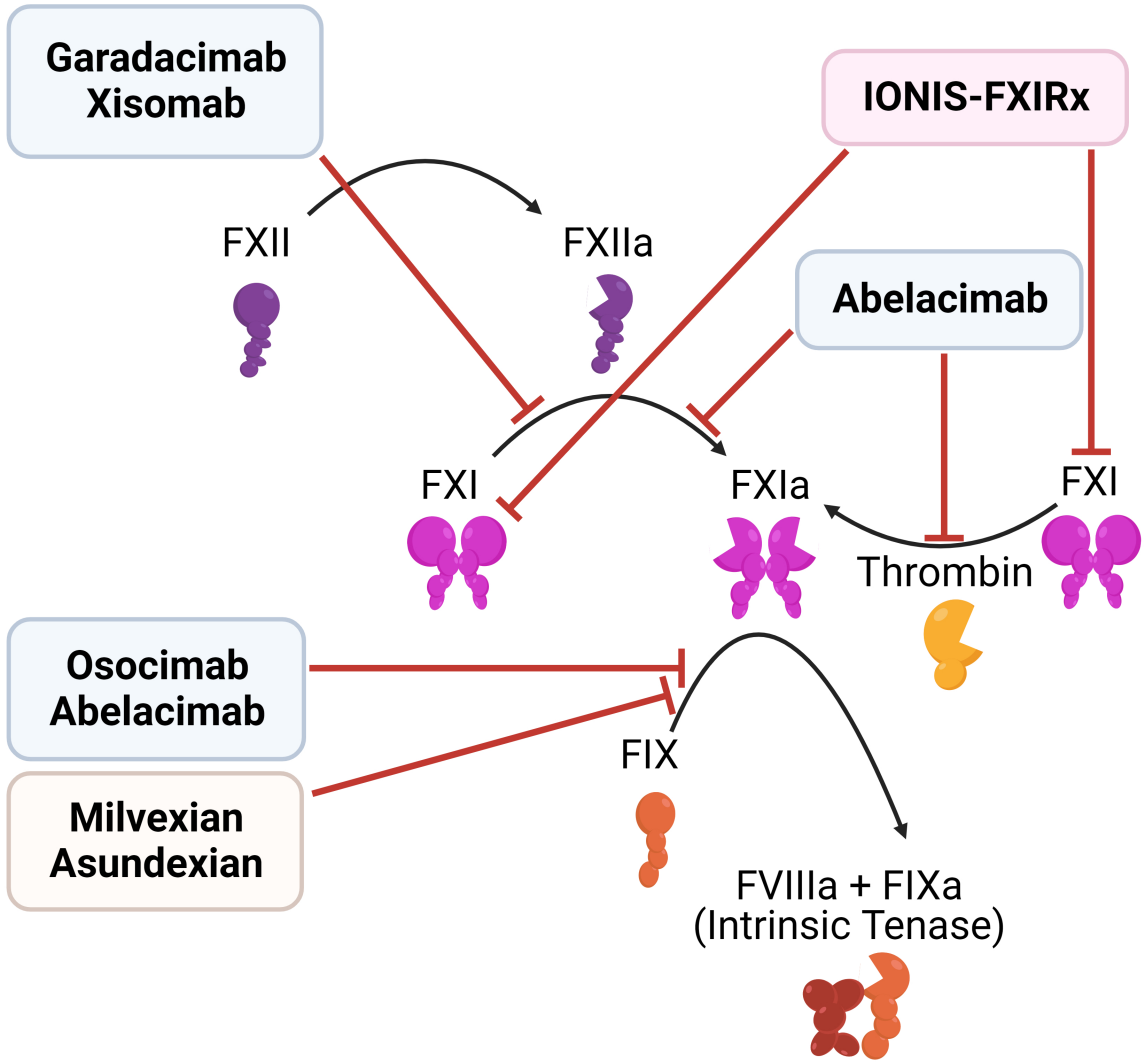
Inhibitors of the contact pathway. Shown are the targets of newly developed inhibitors of the contact system, including antisense oligonucleotides (red), monoclonal antibodies (blue), and small molecule inhibitors (orange).

Multiple strategies have been developed to target FXII and FXI (Figure 6, Table 2):

(1) Antisense oligonucleotides (ASOs): Liver-directed ASOs can be used to selectively knockdown the expression of targeted proteins. ASOs which target components of the contact pathway were first reported in 2010, and have been shown to reduce thrombosis in multiple animal models, with low bleeding risk (153–156). IONIS-FXIRx (also called BAY-2306001, FXI ASO, ISIS 404071, ISIS-416858, and ISIS-FXIRX) was the first of these agents to be tested in humans, when it was tested in a cohort of patients undergoing total knee arthroplasty (157). This subcutaneous FXI-directed ASO reduced FXI levels effectively and lowered VTE risk in patients compared to enoxaparin, with no significant increase in bleeding.
(2) Monoclonal antibodies: Another approach involves parenteral administration of monoclonal antibodies that block clotting factor activation and/or activity. Several antibodies targeting FXI are in development, with differing modes of action: (a) Abelacimab binds the active site of FXI/XIa, locking it in an inactive zymogen-like state. As such, it both prevents the activation of FXI by thrombin or FXII and blocks the activity of FXIa (158, 159). (b) Osocimab binds an allosteric site on FXIa, blocking its activation of FIX (160, 161). Xisomab, inhibits the activation of FXI by FXIIa (162). Garadacimab (formerly CSL 312) is one of the few agents targeting FXII as a monoclonal antibody (163, 164). Unlike ASOs, monoclonal antibodies can be used both in acute and chronic settings, as they have a faster response. ASOs require uptake in the liver and clearance of the existing plasma protein, before their effect may be realized (151).
(3) Small molecule inhibitors: There are both orally and parenterally available small-molecule inhibitors of FXI, such as milvexian (BMS-986177/JNJ-70033093) and asundexian (BAY 2433334), in development. In a Phase II trial of 1242 knee arthroplasty patients, milvexian (25–200 mg) twice daily effectively prevented venous thromboembolism with lower bleeding risk than enoxaparin. Once daily milvexian also showed efficacy in VTE prevention (165). Milvexian is now being assessed in a Phase III trial. Similarly, asundexian at doses of 20 mg and 50 mg once daily demonstrated near-complete *in vivo* FXIa inhibition. These dosages resulted in reduced rates of bleeding, and similar rates of thrombosis, compared to the standard dosing of apixaban (166), suggesting that asundexian is a safer alternative. However, there are concerns about the efficacy of FXIa inhibition, based on these results (152).
(4) Targeting activators of the contact system: In addition to directly targeting the contact pathway, it may also be possible to target components that activate this pathway, such as NETs and polyphosphates. As recently reviewed elsewhere (167), multiple therapeutic strategies are in pre-clinical or clinical stages of development to target NETs, or the generation of NETs. These include approaches to directly or indirectly inhibit the release of nuclear content from neutrophils, the degradation of DNA by DNAse, and the targeting of NET-associated proteins, such as myeloperoxidase. In addition, Baron et al. recently reported that Selinexor, a first-in-class inhibitor of nuclear export approved for use in multiple myeloma patients, effectively prevents NET release *in vitro* (168). Selinexor may not be an effective antithrombotic, however, as it is associated with reduced platelet count, due to an off-target effect on megakaryocytes (169).
  Similarly, polyphosphates may be targeted to prevent thrombosis. La et al. developed a polycationic compounds (termed macromolecular polyanion inhibitors, MPIs) that bind and neutralizes polyphosphates (170). MPIs bind to platelet-released polyphosphates, and reduce thrombus formation in a cremaster muscle laser injury model in mice. In contrast, they did not impair hemostasis in a tail bleeding model.

To summarize, the contact pathway of coagulation is an appealing target for developing safer anticoagulants due to its potential to reduce thrombosis without increasing bleeding risk. Selective FXI or FXII inhibitors have shown promise in preclinical and early clinical studies, and agents that target activators of the contact pathway, such as NETs and polyphosphates are also in development.

## 4. Conclusions

For decades, the “holy grail” of antithrombotic treatment has been a drug that prevents thrombus formation but does not carry a concomitant bleeding risk. This has been a very difficult goal to achieve, as bleeding and thrombosis are closely related processes. Platelets and coagulation factors are required for both, and any compound that broadly inhibits either process has historically carried a bleeding risk. However, exciting progress has been made in recent years, and we may be closer than ever to obtaining the grail. Rapid reversal agents for DOACs have been developed, safer alternatives have been developed to existing antiplatelet and anticoagulant therapeutics, and new targets have been identified that appear to be more specifically involved in the thrombotic process. This is an exciting time for antithrombotic therapy, as we wait to see how effective these new targets and treatment strategies are in clinical trials.
